# Antidepressant-withdrawal mania - a case report

**DOI:** 10.1192/j.eurpsy.2021.543

**Published:** 2021-08-13

**Authors:** M. Magalhães, L. Moreno, R. Mendes, A. Gamito

**Affiliations:** Department Of Psychiatry And Mental Health, Setúbal Hospital Center, Setúbal, Portugal

**Keywords:** Antidepressant-Withdrawal Mania, adverse event of antidepressant discontinuation, bipolar spectrum, hypomania mania

## Abstract

**Introduction:**

An uncommon adverse event of antidepressant discontinuation is the paradoxical withdrawal hypomania or mania. It is rarely described in the literature and its true incidence is unknown, may it be a consequence of underrecognition or misattribution.

**Objectives:**

Alert clinicians of the uncommon Antidepressant-Withdrawal Mania

**Methods:**

Report and discuss, based on online pubmed database, a case of Antidepressant-Withdrawal Mania with Citalopram

**Results:**

A 34 year old woman, with a previous unipolar depressive episode, presents to the emergency department in june/2020 with anxiety, recurrent thoughts of death without a plan, low energy, anhedonia, loss of appetite, sadness and insomnia developing over a period of 7 months. She was prescribed Quetiapine 50 mg XR, Lamotrigine 100 mg, and sent to a psychiatry consultation. After a month and a half there was no important clinical improvement and Citalopram 20 mg was started. The patient reported slow improvement and by august she had a complete symptomatic response. In the beginning of september the patient stopped citalopram abruptly. Three weeks later, she was presented with an irritable mood, increased energy with decreased need for sleep, sweet cravings, easy irritability, racing thoughts, pressure to keep talking and suicidal thoughts. After 2 weeks of Quetiapine 300 mg XR id, Lamotrigine 100 mg id and Olanzapine 5 mg there was a partial symptomatic response.

**Conclusions:**

Antidepressant withdrawal manic states are an under-recognized phenomena, with ill defined patho-physiological pathways and nosology. It is important to continue close follow up of the patient and to investigate whether it can be included on the bipolar spectrum.

